# Low‐Invasive Biomarkers of Canine Mammary Tumours

**DOI:** 10.1002/vms3.70280

**Published:** 2025-03-17

**Authors:** Luo Xinyi, Liu Jinlong, Zhou Bin

**Affiliations:** ^1^ Key Laboratory of Applied Technology on Green‐Eco‐Healthy Animal Husbandry of Zhejiang Province, Zhejiang Provincial Engineering Laboratory for Animal Health Inspection & Internet Technology, Zhejiang International Science and Technology Cooperation Base for Veterinary Medicine and Health Management, China‐Australia Joint Laboratory for Animal Health Big Data Analytics College of Animal Science and Technology & College of Veterinary Medicine of Zhejiang A&F University Hangzhou Zhejiang Province P. R. China

**Keywords:** biomarkers, blood biomarkers, canine mammary tumours, fine needle aspiration cytology, low‐invasive

## Abstract

Canine mammary tumours (CMTs) are the most common type of tumours in older bitches. An early, precise and low‐invasive diagnosis is essential, due to some CMTs being malignant and having a poor prognosis. Fine needle aspiration cytology (FNAC) and blood tests are both low‐invasive diagnostic methods that have been used in veterinary medicine. However, the perfect biomarkers should be identified to diagnose and evaluate the prognosis of CMTs. This review focuses on biomarkers that can be tested by FNA or blood samples based on current literature. Until now, the most studied biomarkers of FNAC, such as Ki‐67, human epidermal growth factor receptor 2 (HER‐2), oestrogen receptor (ER), progesterone receptor (PR), P53, E‐cadherin and cyclooxygenase‐2 (COX‐2). Some common blood biomarkers that have been widely studied include lactate dehydrogenase (LDH), C‐reactive protein (CRP), carbohydrate antigen 15‐3 (CA15‐3) and carcinoembryonic antigen (CEA). The novel biomarkers will also be mentioned: cancer stem cells (CSCs), circulating tumour cells (CTCs), miRNAs and circulating cell‐free DNA (cfDNA); they are all useful markers. Copper ion and serum ferritin (SF) are good markers of human breast cancer; they may be candidates of CMTs biomarkers, too. In conclusion, many biomarkers are suitable for diagnosing and/or prognosing CMTs; combining a couple of them can increase the specificity; more detailed research should be done.

## Graphical abstract

Fine needle aspiration cytology and blood tests are both low‐invasive diagnostic methods that have been used in veterinary medicine. In the FNA samples, an elevated expression of Ki‐67, P53, E‐cadherin or COX‐2, whether observed individually or in combination with a decreased expression of HER‐2, ER or PR, is a strong indicator of an adverse prognosis for canine mammary tumours. In blood tests, apart from miRNA, an elevation in the concentrations of LDH, CRP, CA15‐3, CEA, CSCs, CTCs, cfDNA, copper ions or serum ferritin serves as an indication of a poor prognosis for canine mammary tumours.

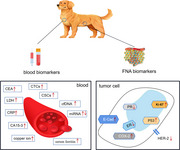

## 1 Introduction

Canine mammary tumours (CMTs) are the most common tumour in intact female dogs (Vazquez et al. 2023). The main malignant types include papillary carcinoma, complex carcinoma, solid carcinoma and adenocarcinoma (Salas et al. 2015), whereas non‐malignant CMTs consist of simple adenomas, benign mixed tumours, ductal papillomas and fibroadenomas. Mammary glands in the same individual may exhibit two or more tumour types.

As CMTs represent different kinds of tumours and different treatment methods may be needed, proper diagnostics are extremely important. Current diagnostic methods encompass blood tests, radiograph or CT of the chest, ultrasound examination of mass and abdomen, fine needle biopsy of mass (for differential diagnosis of other tumours or non‐neoplastic conditions) and regional lymph nodes and histopathological evaluation of the removed mass (Kaszak et al. 2022). Ultrasound techniques can assess mass volume, margins and even the vascular flow (Soler et al. 2016). Blood tests, including complete blood count (CBC), blood smear, biochemistry and blood gases, may offer information on the relationship between mammary tumours and blood parameters, although their specificity is often unsatisfactory. Histological examination post‐excision remains the standard (Chocteau et al. 2019) and final (Soler et al. 2016) diagnostic method, but it is neither an early nor a low‐invasive diagnosis method.

**FIGURE 1:**
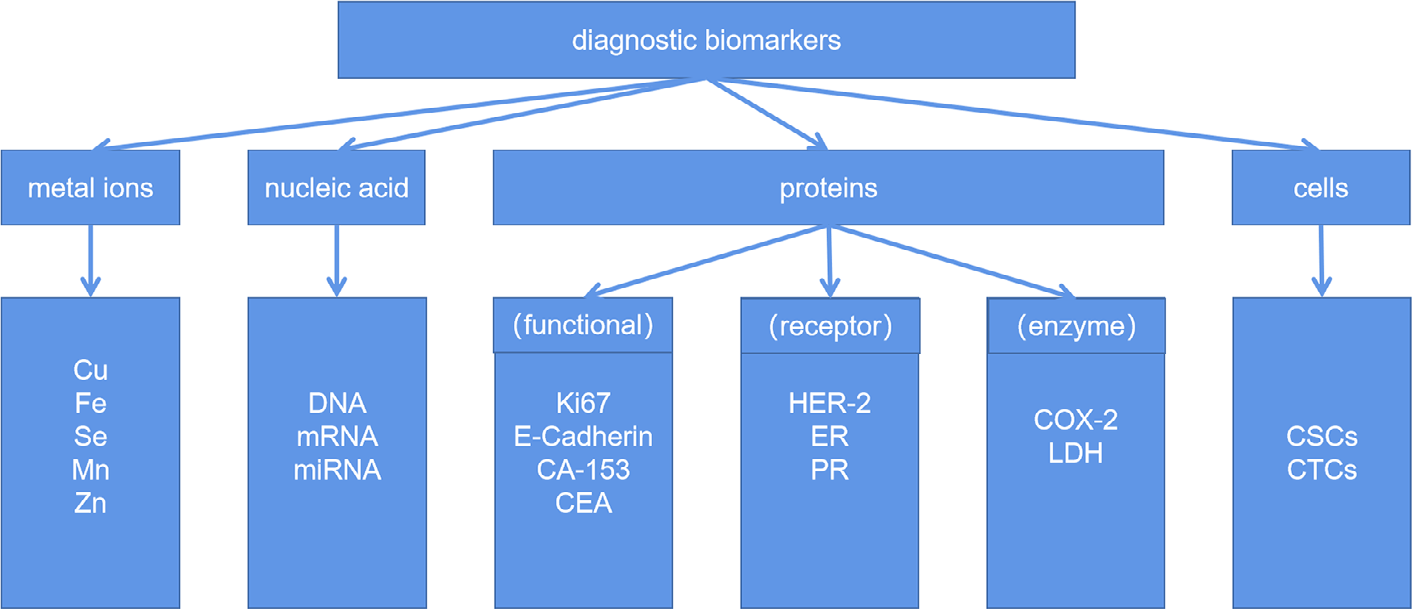
Categories of tumour diagnostic biomarkers. CSC, cancer stem cell; CTC, circulating tumour cell; ER, oestrogen receptor; HER‐2, human epidermal growth factor receptor 2; LDH, lactate dehydrogenase; PR, progesterone receptor.

In contrast to human breast cancer (HBC), CMTs are insufficiently studied, often discovered in advanced stages. Thus, more and more research studies had been focused on the diagnostic approach, treatment method and prognosis evaluation of CMTs. The urgency for early‐ and low‐invasive diagnostic methods is emphasized given the heterogeneous nature of CMTs. Still now, limited studies have explored low‐invasive diagnostic and/or prognosis biomarkers of CMTs. Biomarkers encompassing cells, proteins, nucleic acid and mental ions (Figure 1) can provide information about disease presence, therapeutic effect or further prognosis when measured in blood or tissue samples (Perera et al. 2022). Blood biomarkers can potentially detect malignancies before visible macroscopic changes, aiding confirmation, treatment monitoring and prognosis evaluation.

Fine needle aspiration cytology (FNAC) is a cost‐effective, low‐invasive technique focusing on early tumour detection through morphological examination (Yildirim and Gurel 2012). FNAC offers advantages like easy handling and quick results and is applicable for identifying cellular origins and cytological differentiation of tumour cells (Dolka et al. 2018). One research study indicated that the expression of markers was distinctively related to some cytological parameters in HBC (Li et al. 2022). A study in eastern India, ICC and immunohistochemistry (IHC) were done for ER, PR and human epidermal growth factor receptor 2 (HER‐2) evaluation. Comparing samples from FNAC with samples from biopsy specimens, the ER and PR markers demonstrated very good specificity (86.7% and 94.7%, respectively), sensitivity (83.3% and 88.5%, respectively) and correspondence rates (84% and 91%, respectively). However, for HER‐2, the performance was merely good (specificity 77.4%, sensitivity 71.4%, correspondence rate 76%) (Tripathy et al. 2018). In a prospective study, FNAC samples were obtained from 50 dogs suffering from mammary tumours; results of histopathological and cytological examination were compared. Comparing to histopathological examination, the specificity and sensitivity of cytological evaluation of CMTs are 94%–96% and 65%–88%, respectively (Simon et al. 2009). As cytological examination has satisfactory specificity, sensitivity and accuracy for the diagnosis of malignancy of CMTs, some cytology smears through FNAC were investigated. Above all, the biomarkers of FNAC were nearly the same as histopathological biomarkers, both in HBC and in CMTs.

Although FNA has many advantages in the diagnosis of neoplastic diseases, it still has some limitations. First, due to the small sampling volume, it may be impossible to obtain representative cells. Second, cytological specimens are mainly suitable for ICC, but proper optimization and strict quality control of high‐quality stainings are fundamental. To overcome the issue of small sample size, we can improve the situation by increasing the sampling sites or combining with ultrasound‐guided FNA. Regarding the issue of cytological interpretation, apart from an experienced cytopathologist, cell blocks (CBs), which are created by absorbing more specimens by FNA for ICC and genetic testing, can offer versatility for diagnostic, prognostic and predictive assays (Tani et al. 2017).

Compared to FNAC, blood samples are much more easier to obtain and measure. Furthermore, blood testing can be repeated at short intervals, which allows monitoring the parameters of patients over time. Over the past two decades, many kinds of blood biomarkers were used for diagnosing HBC, such as nucleic acids, proteins and even cells (circulating tumour cells [CTCs], cancer stem cells [CSCs]). Measurement of blood biomarkers in canines for early diagnosis of tumours, assessment of disease progression and evaluation response to chemotherapy and/or radiotherapy represents a milestone (Colombe et al. 2022).

In this review, we mainly describe some promising biomarkers of CMTs by FNAC and blood samples (Table 1). Some of these biomarkers are already in clinical use, whereas others are still under investigation.

**TABLE 1:** The function and main application of different biomarkers.

Biomarker	Sampling location	Function	Main application	References
Ki‐67	Tumour mass	Cell proliferation and apoptosis	A widely used and reliable marker of tumour proliferation and aggressiveness	Pathmanathan and Balleine (2013), Rakha et al. (2022), Li et al. (2015), Nielsen et al. (2021), Aydogan et al. (2018), Dolka et al. (2013), Araujo et al. (2016), Choi and Kim (2010), Carvalho et al. (2016)
HER‐2	Tumour mass	Tumour growth and differentiation	HER‐2 over‐expression is related to higher tumour aggressiveness, poor response to treatment and shorter survival time	Brunetti et al. (2021), Ross et al. (2016), Wolff et al. (2013), Toi et al. (2018), Seung et al. (2020), Ressel et al. (2013), Hsu et al. (2009), Muscatello et al. (2022), Soares et al. (2017), Kaszak et al. (2018), Yang et al. (2023)
PR and ER	Tumour mass	Tumour growth and development	ER^+^ and/or PR^+^ is usually connected with good clinical outcomes	Pastor et al. (2020), Hilton et al. (2018), Yip and Rhodes (2014), Watanabe et al. (2022)
P53	Tumour mass	Cell division and apoptosis	Some studies have shown that over‐expression of P53 was related to shorter overall survival other authors stressed no correlation	Kim et al. (2014), Ochiai et al. (2018), Klopfleisch and Gruber (2009), Munday et al. (2019), Phibey et al. (2009), Lee et al. (2004)
E‐Cadherin	Tumour mass	Cell adhesion	Lower expression of E‐cad can increase the ability of tumour metastasis and invasion	Grayton et al. (2017), Natalia and Jaime (2019), Kaszak et al. (2020), Ieni et al. (2015), Gao et al. (2013), Li et al. (2017), Chetty and Serra (2008), Asproni et al. (2015), Lopuszynski et al. (2015)
COX‐2	Tumour mass	Inflammation, metastases, recurrence	Over‐expression of COX‐2 is associated with more advanced cancer stage, higher risk of tumour recurrence, presence of metastases and poor overall survival rate	Yu et al. (2022), Szweda et al. (2019), Gregorio et al. (2021), Millanta et al. (2016), Anadol et al. (2017), Queiroga et al. (2005), Carvalho et al. (2017), Gregorio et al. (2021), Raposo et al. (2017), Iturriaga et al. (2017), Alkan et al. (2012)
LDH	Blood	Metabolism	Higher level of LDH is related to advanced stage and has significant effect on shorter OS and PFS	Tamura et al. (2015), Deme and Telekes (2017), Liu et al. (2019), Pelizzari et al. (2019), Liu et al. (2015), Di Gioia et al. (2016)
CRP	Blood	Inflammation and recurrence	Concentration of CRP was higher in advanced stages than in early stages	Campos et al. (2012), Pepys (1981), Hart et al. (2020), Elshabrawy et al. (2012), Edimiris‐Herrmann et al. (2022), Chan et al. (2015), Crossley and Coloma (2010), Szczubial et al. (2018)
CA15‐3	Blood, tumour mass	Cell adhesion and metastases	Higher level of serum CA15‐3 was also highly related to local and/or distant metastases	Battisti et al. (2013), Michael et al. (2004), Apostolopoulos et al. (2015), Marchesi et al. (2010), Baba et al. (2019), Bidard et al. (2012), Manuali et al. (2012), Yeganeh‐Amirkande et al. (2015)
CEA	Blood, tumour mass	Cell adhesion and metastases	CEA is closely correlated to metastases and therapeutic responses	Szekanecz et al. (2007), Senhorello et al. (2020), Hao et al. (2019), Hing et al. (2020), Wang et al. (2014), Jain et al. (2021)
CTCs	Blood	metastases	They are highly correlated with distant metastases	Fan et al. (2021), Mu et al. (2015), Cristofanilli et al. (2004), Massimo et al. (2005), Da Costa et al. (2012), Ucmak et al. (2021)
CSCs	Blood	Tumour recurrence	They are highly correlated with recurrence, distant metastases and drug resistance	Marconato et al. (2019), Celia‐Terrassa (2018), Hassan and Seno (2020), Moon and Kim (2021), Gavhane et al. (2016), Magalhaes et al. (2013), Michishita et al. (2012)
mRNAs	Blood, tumour mass	Proliferation, angiogenesis, migration, apoptosis	MiRNAs can regulate all the processes of tumour development	Rogez et al. (2019), Hamam et al. (2017), Cardinali et al. (2022), Fish et al. (2020), Ramadan et al. (2022), Osaki et al. (2016), Rybicka et al. (2015)
Copper ion	Blood	Tumour migration and metastases	Higher level of copper is involved in tumour migration and metastasis, tumourigenesis and angiogenesis	Heishima et al. (2017), MacDonald et al. (2014), Brady et al. (2014), Pan et al. (2002), Ding et al. (2015), Choi et al. (2019), Feng et al. (2012), Lossow et al. (2021)
SF	Blood	Metabolism	Higher SF level is related to tumour malignant behaviour	Askar et al. (2009), Cullis et al. (2018), Alkhateeb and Connor (2013), Chiou and Connor (2018), George et al. (2021), Hu et al. (2019), Ji et al. (2014), Hong et al. (2017), Konz et al. (2014), Kell and Pretorius (2014), Weinstein et al. (1989), Alonso García et al. (2016), Lian et al. (2019)
cfDNA	Blood	Proliferation, angiogenesis	Higher level of cfDNA is related to tumour proliferation and more advanced cancer stage	Anita et al. (2016), Snyder et al. (2016), Wan et al. (2017), Schwarzenbach (2013), Chul (2024), Tagawa et al. (2019), Flory and Wilson‐Robles (2024), Lee et al. (2019), Wilson‐Robles et al. (2022), Kruglyak et al. (2021)

Abbreviations: CEA, carcinoembryonic antigen; cfDNA, cell‐free DNA; COX, cyclooxygenase; CRP, C‐reactive protein; CSC, cancer stem cell; CTC, circulating tumour cell; ER, oestrogen receptor; HER‐2, human epidermal growth factor receptor 2; LDH, lactate dehydrogenase; PR, progesterone receptor; SF, serum ferritin.

## 2 FNAC Biomarker

### 2.1 Ki‐67

The Ki‐67 antigen was originally identified in the early 1980s, encoding two protein isoforms with molecular weights of 395 and 345 kDa (Pathmanathan and Balleine 2013). Ki‐67 protein (pKi67) is a kind of nuclear protein which is expressed during mitosis and can only be detected in the cell nucleus. Its expression is associated with the proliferative activity of malignant tumour cells, making it a widely used marker of tumour proliferation (Rakha et al. 2022) and aggressiveness (Li et al. 2015). The prognostic value of Ki‐67 has been investigated in several studies, with results indicating its reliability as a marker in breast cancer and cancers of other organs.

In a study involving 65 dogs with mammary tumours (carcinosarcoma, tubulopapillary carcinoma and complex carcinoma), Ki‐67 expressions were negative in 49 dogs and weak in 16 dogs, suggesting Ki‐67 may not be an ideal candidate for diagnosing CMTs (Nielsen et al. 2021). However, another research indicated that, compared to other types of CMTs, Ki‐67 expression was significantly correlated with the canine mammary sarcomas (Aydogan et al. 2018). High Ki‐67 index along with other parameters, such as tumour volume, lymph node or distal metastasis, was related to shorter overall survival (OS) and worse prognoses in CMTs (Dolka et al. 2013). A study had been done to compare the correlation coefficient between histological sections and cytological smears. As a marker, Ki‐67 staining was successful in 19/23 (83%) sections and 17⁄24 (71%) smears; the correlation coefficient between the percentage of Ki‐67‐positive cells in histological sections and cytological smears was 0.677 (*p* < 0.01) (Araujo et al. 2016). Another study showed that the proliferation index (PI) of Ki‐67 (both in tumours and adjacent non‐neoplastic mammary glands) was associated with clinico‐pathological features of tumour aggressiveness and shorter OS (Choi and Kim 2010). Whether Ki‐67 can be used in diagnosing CMTs depends on the types of tumours, and it should be evaluated with other parameters (Carvalho et al. 2016); fine needle biopsy of Ki‐67 creates a choice for early tumour diagnosis, and further studies need to be done.

### 2.2 Human Epidermal Growth Factor Receptor 2

In breast cancer, HER‐2, a Type I receptor tyrosine kinase, plays a crucial role in cell proliferation, differentiation, migration and invasion (Brunetti et al. 2021). It acts as a negative prognostic factor. It has been recommended by the American Society of Clinical Oncology (ASCO) for use in treatment monitoring and prognostic evaluation in hormone receptor–positive breast cancer (HBC) (Ross et al. 2016). HER‐2 over‐expression in HBC is associated with higher tumour aggressiveness, poor therapy response and shorter survival time. In breast carcinoma, samples obtained by fine needle aspiration can be used to evaluate the expression of HER (Wolff et al. 2013).

The utility of HER‐2 as a good biomarker in CMTs remains controversial. Techniques like protein expression measurement and gene amplification evaluation are recommended for determining HER‐2 status in HBC and CMTs (Toi et al. 2018). Many research studies have shown that over‐expression of HER‐2 is not related to bad prognosis in canine tumours (Seung et al. 2020); some research studies even demonstrated HER‐2 overexpression is associated with an increased survival rate. In veterinary medicine, the survival rate within 2 years after surgery was higher in dogs with HER‐2‐overexpressing malignant mammary tumours than with the normal level of HER‐2 (Ressel et al. 2013). In another study, a higher copy number of the gene was correlated with HER‐2 protein over‐expression but not with the tumour's biological behaviour (such as tumour size, histological type and gradation, lymph node and distant metastasis) (Hsu et al. 2009). A study with 90 bitches also showed that HER‐2 over‐expression in the tumours has no connection with lymph node metastasis and/or with distant metastasis (Muscatello et al. 2022). On the contrary, many research studies showed that higher HER‐2 expression was related to higher tumour mitotic index, bigger tumour size, higher histological grade and worse prognosis (Soares et al. 2017). Compared with other types of mammary tumours, HER‐2 positive mammary carcinoma progresses more rapidly and exhibits a higher degree of malignancy (Kaszak et al. 2018). In both Hormone Receptor‐Positive Breast Cancer (HBC) and CMTs, tumours that are ER‐/PR‐/HER‐2‐ are called triple‐negative tumours. In veterinary medicine, almost all triple‐negative tumours are found at advanced clinical stages and are associated with peritumoural and vascular invasion (Yang et al. 2023). Thus, HER‐2 remains a widely used diagnostic biomarker for CMTs, but more studies need to be conducted.

### 2.3 Hormone Receptors

Progesterone receptor (PR) and oestrogen receptor (ER) are types of hormone receptors. These receptors play a crucial role in cellular function as they can modulate cellular gene expression through the activation or inhibition of the transcription process. They are members of the nuclear receptor family of evolutionarily related ligand‐activated transcription factors, which are crucial regulators of normal breast development and function and also play an important role in HBC (Pastor et al. 2020). ER and PR are extensively studied markers in both HBC and CMTs. In breast carcinoma, samples obtained by fine needle aspiration can be used to evaluate the expression of ER and PR (Wolff et al. 2013). Studies have shown that ER and PR are reliable markers for diagnosing and predicting prognosis of HBC (Hilton et al. 2018). Many studies have proved that the expression of ER and/or PR is more common in benign tumours compared to malignant tumours, and ER^+^ and/or PR^+^ is typically associated with favourable clinical outcomes in CMTs (Yip and Rhodes 2014). Research suggests that spaying during mastectomy in bitches with high serum oestrogen levels can lead to a longer lifespan for ER^+^ tumours. ER^+^/PR^+^ tumours exhibit a better prognosis compared to ER^−^/PR^+^ tumours, with ER^−^/PR^−^ tumours having the least favourable prognosis (Watanabe et al. 2022). In CMT, ER and PR are very good biomarkers (both FNAC and blood biomarkers), and their status can influence the treatment options. Despite current findings, more research is essential to deepen our understanding of the role of ER and PR in HBC and CMTs.

### 2.4 P53

The *p53*, which was a tumour suppressor, plays a crucial role in tumour development. The p53 protein, as an initiator of cell cycle arrest and apoptosis, can inhibit cell proliferation. Notably, mutations in the *p53* gene are prevalent, being discovered in over half of human cancers, and have also been identified in various canine cancers (Kim et al. 2014). In veterinary medicine, the mutation status of *p53* and expression level of P53 as prognostic markers are still controversial: Some studies have shown that over‐expression of P53 was related to shorter OS (Ochiai et al. 2018); other authors stressed no correlation (Klopfleisch and Gruber 2009). In CMTs, compared to benign tumours, the expression level of P53 was much higher in malignant tumours (Munday et al. 2019). The increase in P53 is greater in higher grade tumours with higher proliferation rates. A significant correlation between increases in P53 expression and shorter OS time has also been found (Phibey et al. 2009). Furthermore, the expression level of P53 was found to be higher in large‐breed dogs than in small‐breed dogs, which could suggest some breed predispositions (Ochiai et al. 2018). Beyond its diagnostic utility, P53 also emerges as a therapeutic target (Lee et al. 2004), offering further avenues for research and treatment in veterinary oncology.

### 2.5 E‐Cadherin

As a group of transmembrane glycoproteins, cadherins can regulate adhesions of cell to cell during embryogenesis, tissue morphogenesis, differentiation and carcinogenesis. The classical cadherins, including epithelial‐cadherin (E‐cad), placental‐cadherin (P‐cad) and neural‐cadherin (N‐cad) (Grayton et al. 2017). E‐cad, specifically associated with epithelial cell adhesion, plays a pivotal role in carcinogenesis (Natalia and Jaime 2019). In the study of human pancreatic cancer, samples obtained through fine needle aspiration can be examined by both ICC (Kaszak et al. 2020) and IHC (Ieni et al. 2015). Lower expression of E‐cad is a phenomenon of epithelial–mesenchymal transition (EMT), a phenomenon enhancing tumour metastasis and invasion. Therefore, reduced expression of E‐cad corresponds to a higher tumour histological grade, larger tumour volume, more lymph node metastasis and indicates poor disease outcomes (Gao et al. 2013). A meta‐analysis showed that lower expression of E‐cad might be a predictor of a worse prognosis and might be a therapy target for treating HBC (Gao et al. 2013).

In veterinary medicine, studies show that the cytoplasmic and nuclear location of E‐cad, rather than its expression levels, is linked to the downregulation of its tumour suppressor role (Li et al. 2017). In a study of canine mammary lesions, 54 samples (15 hyperplasias, 7 adenomas, 32 carcinomas) were submitted to immunohitochemical test, the expression of E‐cad were 100% (hyperplastic), 86% (adenomas) and 34% (carcinomas). The results demonstrated that in dogs bearing tumours, those with tumours showing positive staining for both E‐cad and PTEN exhibited a longer OS and experienced less frequent lymph node invasion (Chetty and Serra 2008). Another study suggested that depending on the histological type and malignancy grade, lower E‐cad expression was observed in malignant tumours when compared to normal mammary glands and benign mammary tumours (Asproni et al. 2015). A study in 2022 showed that mRNA expression of E‐cad was lower in malignant tumours than in healthy dogs (Lopuszynski et al. 2015). So, E‐cad maybe a negative biomarker of CMTs.

### 2.6 Cyclooxygenase‐2 (COX‐2)

Cyclooxygenase (COX), which can catalyse arachidonic acid to prostanoids, is an enzyme belonging to the myeloperoxidase family. These contain prostaglandins, thromboxane and prostacyclin, which are bioactive proteins that can regulate a variety of physiological processes both in animals and humans (Yu et al. 2022). COX has three isoforms: COX‐1, COX‐2 and COX‐3. COX‐1, which is responsible for different kinds of physiological activity, can be detected in normal tissues and organs; COX‐2 is nearly undetectable in normal tissues; it is produced in tissues due to inflammatory reactions, tumourigenesis and other abnormal processes (Szweda et al. 2019); COX‐3, of few reports, is expressed mainly in the aortic wall and the central nervous system (Yu et al. 2022).

The involvement of COX‐2 in tumourigenesis is complex and not fully understood. Over‐expression of COX‐2 is linked to advanced cancer stage, higher recurrence risk, presence of metastases and poor OS rate in human cancers. Some researchers (Gregorio et al. 2021) discovered that the positive rate of COX‐2 expression was 18% (4/22) in non‐neoplastic mammary gland tissue, 83% (30/36) in canine mammary carcinomas and 20% (2/10) in adenomas. Another study showed that the mRNA level of COX‐2 was significantly higher in mammary tumours (both malignant and benign) than in adjacent non‐neoplastic tissue. The levels of COX‐2 (mRNA) were related to the histological grade of malignancy in CMTs, being highest in Grade 3 tumours, higher in Grade 2 tumours and lowest in Grade 1 tumours (Millanta et al. 2016). A study (Anadol et al. 2017) assessed the expression level of COX‐2 in CMTs to evaluate whether it has any connection with clinical and/or pathological parameters and the prognostic significance. In that study, especially in inflammatory mammary carcinomas, direct proportional relationships were discovered between the expression of COX‐2 and many clinical and/or pathological parameters; higher level COX‐2 were referred to larger tumour volume, ulceration of skin, malignant histological type, shorter time of metastases and/or relapses, shorter OS and shorter PFS. In a multivariate survival study, high COX‐2 expression was associated with increased angiogenesis, proliferation and tumoural inflammatory infiltrate in canine malignant mammary tumours (Queiroga et al. 2005). Another study also showed that COX‐2 can be a negative prognostic factor in canine tumours (mammary carcinoma, mast cell tumour, melanoma, osteosarcoma and renal carcinoma) (Carvalho et al. 2017). In a study of canine inflammatory mammary cancer (CIMC), gene expression of COX‐2 was higher in CIMC than in no‐CIMC types (Gregorio et al. 2021). Therefore, many research studies, both in vivo and in vitro, have been done to treat CMTs with specific COX‐2 inhibitors, for example, meloxicam (Raposo et al. 2017), piroxicam and deracoxib (Iturriaga et al. 2017) and celecoxib (Alkan et al. 2012), and many of them had the desired effect.

There are many research studies supporting the vital function of COX‐2 in CMTs, especially in inflammatory carcinomas; whether it can be a good biomarker in other types of tumours, more research studies are needed to explore its significance.

## 3 Blood Biomarkers

### 3.1 Lactate Dehydrogenase (LDH)

Normal cells metabolize glucose through glycolysis and oxidative phosphorylation, whereas malignant tumour cells exhibit a distinct metabolic profile, relying more heavily on glycolysis. In anaerobic conditions, glycolysis can provide the fastest speed of energy needed for cell proliferation. An essential enzyme in glycolysis, lactate dehydrogenase (LDH), plays a significant role in this process by catalysing the conversion of pyruvate to lactate, a reversible reaction. LDH isoenzyme, which was expressed mainly in malignant tumour cells, can increase the formation of lactate (Tamura et al. 2015). Nowadays, many research studies have evaluated the prognostic significance of serum LDH in HBC, but the results remain unclear. A meta‐analysis involving 6102 patients demonstrated that elevated LDH levels significantly correlated with shorter OS and PFS (Deme and Telekes 2017). In a study of 392 women with metastatic breast cancer (MBC), elevated serum LDH concentrations were associated with a worse prognosis; LDH levels and their variation during first‐line treatment were identified as potential predictors for OS and PFS in MBC (Liu et al. 2019). Another study highlights the prognostic value of serum levels of LDH and other parameters (albumin, total bilirubin) in non‐metastatic HBC (Pelizzari et al. 2019). A retrospective analysis in 2016 suggested that certain biomarkers, including LDH, could enhance the diagnostic sensitivity of carbohydrate antigen 15‐3/carcinoembryonic antigen (CA15‐3/CEA) without compromising diagnostic specificity (Liu et al. 2015).

A remarkable increase of serum LDH was observed in bitches with malignant mammary tumours, too. One study showed that, higher levels of serum LDH showed significant positive correlation with advanced stage in CMTs (Di Gioia et al. 2016). In another study, many blood parameters were compared between 6 healthy bitches and 12 bitches suffering from mammary tumours; the activity of LDH was significantly higher in the latter. Serum LDH concentration can act as a biomarker for HBC and CMTs; much more data are needed to update current findings.

### 3.2 CRP (C‐Reactive Protein)

C‐reactive protein (CRP), a kind of acute‐phase reactive proteins (APPs), is a sensitive and widely used systemic marker of inflammation and tissue damage. Primarily synthesized in the liver, its production is triggered by cytokines like TNF‐α, IL‐1 and IL‐6. Elevated CRP levels are associated not only with infections or tissue damage but also with various diseases, including autoimmune, rheumatic and cardiovascular diseases, arteriosclerosis and cancers (Campos et al. 2012). Elevated CRP levels are closely relevant to reduced OS, shorter PFS and higher risk of death in patients suffering from tumours (Pepys 1981). A 2012 study demonstrated a strong association between increased CRP concentration and higher risks of cancer incidence, recurrence and death, suggesting CRP as a consistent indicator of cancer risk compared to other markers (Hart et al. 2020). A retrospective analysis in 152 women with breast cancer revealed a worse prognosis in those with higher CRP concentrations after neoadjuvant chemotherapy (Elshabrawy et al. 2012). A meta‐analysis further established a dose–response relationship between CRP concentration and cancer risk in HBC (Edimiris‐Herrmann et al. 2022).

In a veterinary study, 30 bitches were used to investigate CRP level in benign and malignant mammary tumours; result showed that the probability of a mammary tumour being malignant was 61% or even much higher when CRP values ≥8 mg/L (Chan et al. 2015). A 2018 study with 53 female dogs (10 benign tumours, 33 malignant tumours and 10 healthy dogs) explored the relationship between CRP concentration and clinical characteristics of tumours. Findings suggested that in malignant tumours, CRP concentration was higher in advanced stages than in early stages, emphasizing CRP as a valuable biomarker in malignant CMTs (Crossley and Coloma 2010). Another study revealed significantly higher serum CRP concentrations in dogs and bitches with metastatic and malignant tumours compared to healthy counterparts and those with benign tumours (Szczubial et al. 2018). If CRP is to be used as a biomarker for CMTs, it is crucial to exclude any underlying infection conditions.

### 3.3 Carbohydrate Antigen 15‐3

CA15‐3, a transmembrane glycoprotein encoded by the *MUC1* gene (Battisti et al. 2013), plays a role in tumour development by promoting proliferation, survival, dissemination and adhesion of tumour cells through involvement in immunosuppression (Michael et al. 2004). In HBC, CA15‐3 and CEA are widely used blood biomarkers. They are recommended by EGTM (European Group on Tumor Markers) and ASCO to assist in prognostic evaluation and treatment monitoring of HBC (Liu et al. 2019). Many methods had been developed to measure the concentration of serum CA15‐3, for example, the direct chemiluminescence kit utilized in human medicine (Apostolopoulos et al. 2015), canine‐specific kits for CA15‐3 (Bioassay Technology Laboratory) (Marchesi et al. 2010) and commercial noncompetitive ELISA immunoassay kits (Liu et al. 2019). In a prospective observational study, CA15‐3 demonstrated common elevation in metastatic breast cancers, indicating strong prognostic value (Baba et al. 2019).

In veterinary medicine, studies have consistently shown significantly increased CA15‐3 concentrations in dogs with mammary tumours compared to healthy ones (Apostolopoulos et al. 2015). Although no significant differences were found between the CA15‐3 concentration and patient age, tumour numbers, sizes or histological types, a close relationship was observed with tumour grade (Bidard et al. 2012). Higher serum CA15‐3 levels were strongly associated with local and/or distant metastases, particularly in cases involving lymph node involvement (Di Gioia et al. 2016). However, the higher concentration of CA15‐3 is not specific to tumours, as an increased concentration of CA15‐3 can also be found in hepatitis (Manuali et al. 2012) and arthritis (Yeganeh‐Amirkande et al. 2015). If CA15‐3 is used as a biomarker of CMTs, some specific benign conditions (chronic renal failure, hepatitis and even dermatological diseases) should be excluded. In diagnosing CMTs, it is a promising biomarker, but in order to increase the diagnostic specificity and sensitivity, it is advisable to detect CA15‐3 simultaneously with other biomarkers, such as CEA.

### 3.4 Carcinoembryonic Antigen

CEA, another glycoprotein, is responsible for the adhesion of intracellular. It's primarily produced by normal gastrointestinal mucosa cells, exhibiting a molecular weight of approximately 200 kD. Over‐expression of CEA is observed in various human adenocarcinomas, such as colon, rectum, breast and lung cancers (Szekanecz et al. 2007). CEA serves as a broad‐spectrum tumour marker, notably aiding in the diagnosis of adenocarcinoma (Senhorello et al. 2020). In metastatic HBC, the concentration of CEA is closely correlated to therapeutic responses (Hao et al. 2019). Expect for monitoring tumourigenesis and therapeutic responses, it is also used to determine the prognosis in human medicine (Hing et al. 2020).

Despite promising research results, CEA's application in veterinary medicine remains limited. In a study, 60 dogs suffering from mammary tumours were divided into 3 groups: those with benign tumours, those with malignant tumours and control/healthy dogs. The concentration of serum CEA was found to be highest in dogs with malignant tumours, higher in dogs with benign tumours and lowest in healthy dogs (Wang et al. 2014). Another study indicated the sensitivity, accuracy and specificity of CEA in diagnosing malignant CMTs to be 44.6%, 68.1% and 84.1%, respectively (Jain et al. 2021). CEA can be detected through various diagnostic techniques in both tissue and serum samples. qPCR analysis revealed higher CEA mRNA levels in bitches with malignant tumours compared to healthy ones (Lopuszynski et al. 2015). As mentioned above, the evaluation of CEA is more reliable when it is detected in combination with CA15‐3 and other biomarkers.

### 3.5 Circulating Tumour Cells

CTCs are cells that have shed from primary tumours or metastases, circulate in the blood and possess genetic or antigenic characteristics of specific tumours (Fan et al. 2021). They are highly correlated with distant metastases, and thus, they may be vital diagnostic and prognostic biomarkers. In 2004, Cristofanilli et al. (2004) reported that CTC detection in blood by the CellSearch system (Veridex, Raritan NJ, USA) was a prognostic marker of tumours (Mu et al. 2015); moreover, the PFS was related to changes of CTC numbers following one cycle of chemotherapy.

Only a few research studies have been done to focus on CTCs in veterinary medicine so far (Massimo et al. 2005). Blood samples of 9 healthy female dogs and 45 female dogs suffering from malignant epithelial tumours were compared in Turkey. The results showed that EGFR, CLDN7 and EPCAM markers were measurable, and they can be used to provide valuable information on CMTs in terms of clinical pathophysiology (Da Costa et al. 2012). Another study in Germany showed that six mRNA markers (SLC1A1, IRX3, IRX3, CRYAB, ATP8B1 and AGR2) may be suitable for detection of CTCs in CMTs (Massimo et al. 2005).

Disseminated tumour cells (DTCs) are predictive biomarkers in the bone marrow, which can be used to guide therapy in HBC. A research study on canine metastatic mammary carcinoma showed that the percentage of at least 1 CTC per 7.5 mL in peripheral blood was 44.4% (12/27), and at least 1 DTC per 1 mL in bone marrow was 78.6% (11/14). Furthermore, the levels of CTCs/DTCs and the prevalence of positive dogs closely resemble results obtained through CellSearch assay (mentioned above) in metastatic mammary tumour patients at diagnosis (Ucmak et al. 2021). CTCs are good and promising markers; much more research studies should be done to increase the sensitivity, specificity and accuracy.

### 3.6 Cancer Stem Cells

CSCs refer to tumour cells that possess stem cell‐like properties. They commonly have an elevated capacity to initiate new tumours, a long lifespan and drug‐resistance ability (Marconato et al. 2019). The identification and isolation of CSCs present new opportunities for diagnostic and therapeutic strategies. Numerous studies have demonstrated that even in solid tumours, CSCs can differentiate into haematopoietic cells and circulate in the bloodstream (Celia‐Terrassa 2018). So, CSCs will be good markers for the early diagnosis of solid tumours, including HBC and CMTs. In humans, plenty of cell surface antigens, such as CD24, CD44, CD133, ALDH and EPCAM, are used to identify CSCs. The CD44^+^/CD24^−/low^ phenotype of CSCs, which was evaluated commonly, has been found to be associated with a poor prognosis in HBC (Hassan and Seno 2020) and CMTs (Moon and Kim 2021). In one study, IHC was used to detect CSCs; results showed that CD44^+^/CD24^−^ phenotype cells were positively correlated with Grades II and III tumours 4 (Gavhane et al. 2016). An in vitro study of canine mammary carcinoma cell lines showed that ALDH^high^ cells exhibited CSC‐like properties. A total of 104 ALDH^high^ cells were sufficient for cloning tumours in immunodeficient mice through subcutaneous injection (Magalhaes et al. 2013). Conversely, another study involving 96 canine mammary carcinomas, which used IHC, indicated that a higher number of CD44^+^/CD24^−^ cells were associated with a better prognosis rather than a poor prognosis. This suggests that this specific cell phenotype may not be suitable for detecting CSCs in CMTs (Michishita et al. 2012). An in vitro study of canine mammary carcinoma cell lines showed that ALDH^high^ cells showed CSC‐like properties; 10^4^ ALDH^high^ cells were sufficient for cloning tumours in immunodeficient mice through subcutaneous injection (Magalhaes et al. 2013). Conversely, another study involving 96 canine mammary carcinomas, using IHC, indicated that a higher number of CD44^+^/CD24^−^ cells were associated with a better prognosis rather than a poor prognosis. This suggests that this specific cell phenotype may not be suitable for detecting CSCs in CMTs (Michishita et al. 2012).

Currently, one of the most prevalent methods for detecting CSCs is flow cytometry. However, it comes with drawbacks, such as being expensive, time‐consuming and inconvenient. Although CSCs hold promise as biomarkers for diagnosing CMTs, the heterogeneity and multi‐phenotype nature of CSCs pose challenges, requiring further research efforts.

### 3.7 miRNAs

MiRNAs are short, single‐stranded RNA sequences that control posttranscriptional gene expression in a variety of physiological and pathological processes (Rogez et al. 2019). MiRNAs have attracted much attention in human oncology medicine (Hamam et al. 2017). As far as we know, cellular proliferation, differentiation and apoptosis processes are crucial in cancer development; some miRNAs can regulate all these processes. MiRNAs mainly contain two types: oncogenic miRNA (oncomiR) and tumour suppressor miRNA; there is a negative regulation between them (Wang et al. 2014).

Research studies evaluating miRNAs in CMTs are still rare. In a retrospective study, 10 healthy female dogs (5 spayed, 5 intact) and 10 dogs suffering from mammary carcinoma were enrolled. The serum miRNA was evaluated, comparing to healthy dogs; the expression of circulating miRNAs in dogs with mammary carcinoma is differentially, serum miR‐19b represents a candidate biomarker for diagnosis and miR‐18a represents candidate biomarker for prognosis (Cardinali et al. 2022). Another study showed that, compared to CA15‐3, miR‐21, which is released from mesenchymal cells, may be a more sensitive biomarker for CMTs (Fish et al. 2020). A similar research study disclosed that miR‐21 upregulation and miR‐29b (a tumour suppressor miRNA) downregulation in CMTs are related to advanced clinical stage, and they are good biomarkers for diagnosis and prognosis assessment of tumours (Wang et al. 2014). A few studies evaluated the expression profiles of miRNA in CMT cells in vitro, and many miRNAs may be candidates for CMT diagnostic biomarkers. In one study, the expression levels of miRNA‐138a showed the greatest decrease, and miRNA‐143 showed the greatest increase in CMTs (Ramadan et al. 2022). Another study showed that expression level was increased in 9 mRNAs and decreased in 24 miRNAs in CMTs. Moreover, TGF‐β pathway, as potentially altered, was identified through miRNA target prediction and signalling pathway analysis (Osaki et al. 2016). RT‐qPCR was performed to obtain the profiles of circulating serum miR‐214 and miR‐126 in a total of 181 cases of canine tumours and healthy controls. The result showed that miR‐126 was high in most types of tumours, but miR‐214 maybe a good diagnostic biomarker, as it was relatively high in sarcomas (Rybicka et al. 2015).

Therefore, miRNAs are promising markers for the diagnosis of CMTs and other kinds of tumours. Multiple miRNAs maybe good markers for evaluating prognosis and metastatics of tumours, too. In addition, miRNA‐based therapy seems to be very promising for the regulatory function of miRNAs.

### 3.8 Copper Ion

Though many studies have been disclosed copper's involvement in tumour migration and metastasis (Heishima et al. 2017), tumourigenesis (MacDonald et al. 2014) and angiogenesis (Brady et al. 2014) in HBC, only a few works have been done in veterinary medicine. In a study, 84 healthy controls and 88 breast cancer patients were included; concentrations of 15 trace elements in the serum were detected, and 5 (Cu, Cd, Mg, Co and Li) of them were discovered to be significantly higher in breast cancer patients (Pan et al. 2002). A similar study had been done in Korea; seven trace elements were investigated in people with or without breast cancer, and the levels of serum Cu were significantly higher in breast cancer patients with distant metastasis (Ding et al. 2015). Another study in HBC showed that except for Zn, levels of Cu, Mg, Mn, Fe and Se were similar among subgroups of different clinical stages (Choi et al. 2019). Trace elements in cancer patients versus matched controls were compared; cancer patients had elevated serum Cu levels in breast cancer, lung cancer, prostate cancer and colorectal cancer (Feng et al. 2012). There was only one similar research study in veterinary medicine; a total of 15 dogs with malignant mammary tumours and 15 clinically healthy female dogs were included in this study; dogs with tumours exhibited a significant increase in Cu, Fe and Zn (Lossow et al. 2021).

Above all, the level of serum Cu maybe a good marker in human cancers; whether Cu can be a biomarker of CMTs, much more research studies should be done.

### 3.9 SF (Serum Ferritin)

Ferritin is a soluble iron storage protein, which can store bio‐available iron in cells (Askar et al. 2009). In addition to the intracellular form, ferritin can also be detected in circulation, which is termed serum ferritin (SF). SF levels are higher in patients with iron‐overload disease, hemochromatosis and chronic and acute inflammation (Cullis et al. 2018). SF, a multi‐functional protein, can also act as a marker in many neurological diseases (Alkhateeb and Connor 2013) and cancers such as breast cancer (Chiou and Connor 2018), head and neck squamous cell carcinoma (George et al. 2021), lung carcinoma (Hu et al. 2019) and non‐metastatic colorectal cancer (Ji et al. 2014). Apoferritin forms a roughly spherical cage containing 24 subunits of two types, light (L) and heavy (H), in which ferric iron could be stored in the form of iron nanoparticles (Hong et al. 2017). H‐ferritin is highly expressed in breast tissues (Konz et al. 2014); however, elevated levels of L‐ferritin can be detected in tumour lysates, and this elevation correlates with shorter survival time and advanced histological grade (Kell and Pretorius 2014). A study in breast cancer tumour cells showed that Fe:ferritin ratios could be a more precise marker than single iron or ferritin determination in providing information on the malignancy of these cells (Weinstein et al. 1989). One study disclosed that SF concentration was higher in patients with breast mass (who underwent surgery) than in healthy volunteers and benign breast diseases (Alonso García et al. 2016). Another study showed that higher concentrations of serum iron and transferrin saturation but not SF are associated with cancer outcomes (Lian et al. 2019).

SF was detected through ELISA and qRT‐PCR in 178 dogs with CMTs and healthy dogs; results showed that SF levels were highest in the malignant tumour group, higher in the benign tumour group and lowest in healthy control group. Furthermore, combined detection of SF and CA15‐3, CEA may improve the detection sensitivity of CMTs (Jain et al. 2021). Nevertheless, this field requires more research.

### 3.10 Circulating Cell‐Free DNA (cfDNA)

cfDNA refers to fragments of DNA that are found outside of cells and freely circulating in the bloodstream or other body fluids (Anita et al. 2016). If a tumour exists in the body, the subset of cfDNA fragments released by the tumour is called ‘circulating tumour DNA’ or ‘ctDNA’ (Snyder et al. 2016). In recent years, measuring cfDNA in plasma has gained attention as a biomarker in some human tumours, such as breast tumours (Wan et al. 2017). cfDNA can serve as a marker for the diagnosis of many diseases in dogs, including pancreatitis (Schwarzenbach 2013) and various tumours (Chul 2024). The identification of cancer biomarkers, such as genomic alterations in ctDNA or an increase in the quantity of tumour‐associated proteins or cfDNA in a blood sample, indicates the probable presence of cancer in the body (Tagawa et al. 2019). A study conducted in 2019 showed that the methylation of LINE‐1 in cfDNA serves as a liquid biopsy biomarker for both HBCs and dog mammary tumours (Flory and Wilson‐Robles 2024). In another study, the nucleosome concentrations of 528 dogs with various malignancies were compared with those of 134 healthy dogs. The sensitivity of increased circulating nucleosome concentrations for detecting cancer in all dogs was 49.8%, with a specificity of 97% (Lee et al. 2019). In one study, blood samples were taken from 11 dogs with different types of cancer and 5 cancer‐free dogs. All samples were analysed using next‐generation sequencing, and the results showed that genomic alterations could be identified in most dogs bearing tumours (Wilson‐Robles et al. 2022). Another study showed that the cfDNA fragments and integrity index significantly differentiated neoplastic from non‐neoplastic dogs (*p* < 0.05) and enabled the distinction between benign and malignant lesions (Kruglyak et al. 2021). All the results provide evidence that cfDNA can act as a potential application in diagnostic procedures.

## 4 Conclusions

Up to now, the most extensively studied and reliable biomarkers of CMTs are Ki‐67, HER‐2, E‐cadherin, ER, PR and COX‐2. In order to enhance the accuracy of diagnosis, two or more FNAC markers should be tested in combination, and they may even be combined with blood biomarkers. CRP, LDH, CA15‐3 and CEA have been widely investigated in veterinary medicine, and numerous novel biomarkers, such as cancer CSCs, CTCs, miRNAs and cfDNA, have been explored for diagnosing CMTs. CSCs are not only diagnostic markers but also good markers that help in selecting therapeutic protocols, as they show resistance to chemotherapy and/or radiotherapy. CTCs may serve as good markers for tumour metastasis in CMTs; yet, a universal and effective testing method is lacking. Given their high specificity and sensitivity, miRNAs are the most promising biomarkers, with some of them being oncogenes and others being tumour suppressor genes. Because different tumour cell subclones may all release DNA fragments into the blood, cfDNA can serve as an ideal tumour biomarker as it can overcome the influence of tumour heterogeneity on diagnosis. Alterations in trace element concentrations have been observed in both HBC and CMTs, and investigations have indicated that the most useful element for cancer diagnosis is copper ion. Additionally, iron (Fe) and SF may also be candidate biomarkers.

However, most studies were conducted in small population samples; some results may not be truly reliable. So far, most of the biomarkers mentioned above seem to have diagnostic and/or prognostic value in CMTs, but more and more in‐depth research studies are needed in the future.

## Author Contributions

Luo Xinyi was responsible for writing the original draft. Liu Jinlong was responsible for data curation. Zhou Bin was responsible for writing (review and editing) and supervision.

## Ethics Statement

The authors have nothing to report.

## Conflicts of Interest

The authors declare no conflicts of interest.

### Peer Review

The peer review history for this article is available at https://www.webofscience.com/api/gateway/wos/peer‐review/10.1002/vms3.70280.

## Data Availability

The data that support the findings of this study are available from the corresponding author upon reasonable request.
